# Identification of oral therapeutics using an AI platform against the virus responsible for COVID-19, SARS-CoV-2

**DOI:** 10.3389/fphar.2023.1297924

**Published:** 2023-12-22

**Authors:** Adam Bess, Frej Berglind, Supratik Mukhopadhyay, Michal Brylinski, Chris Alvin, Fanan Fattah, Kishor M. Wasan

**Affiliations:** ^1^ Department of Computer Sciences, Louisiana State University, Baton Rouge, LA, United States; ^2^ Department of Environmental Sciences, Center for Computation & Technology, Coastal Studies Institute, Louisiana State University, Baton Rouge, LA, United States; ^3^ Department of Biological Sciences, Louisiana State University, Baton Rouge, LA, United States; ^4^ Department of Computer Science, Furman University, Greenville, SC, United States; ^5^ Department of Urologic Sciences, Faculty of Medicine and the Neglected Global Diseases Initiative, University of British Columbia, Vancouver, BC, Canada

**Keywords:** artificial intelligence, infectious diseases, antiviral peptides, siamese networks, COVID-19, oral therapeutics, SARS-CoV-2, antiviral activity

## Abstract

**Purpose:** This study introduces a sophisticated computational pipeline, *eVir*, designed for the discovery of antiviral drugs based on their interactions within the human protein network. There is a pressing need for cost-effective therapeutics for infectious diseases (e.g., COVID-19), particularly in resource-limited countries. Therefore, our team devised an Artificial Intelligence (AI) system to explore repurposing opportunities for currently used oral therapies. The eVir system operates by identifying pharmaceutical compounds that mirror the effects of antiviral peptides (AVPs)—fragments of human proteins known to interfere with fundamental phases of the viral life cycle: entry, fusion, and replication. eVir extrapolates the probable antiviral efficacy of a given compound by analyzing its established and predicted impacts on the human protein-protein interaction network. This innovative approach provides a promising platform for drug repurposing against SARS-CoV-2 or any virus for which peptide data is available.

**Methods:** The *eVir* AI software pipeline processes drug-protein and protein-protein interaction networks generated from open-source datasets. *eVir* uses Node2Vec, a graph embedding technique, to understand the nuanced connections among drugs and proteins. The embeddings are input a Siamese Network (SNet) and MLPs, each tailored for the specific mechanisms of entry, fusion, and replication, to evaluate the similarity between drugs and AVPs. Scores generated from the SNet and MLPs undergo a Platt probability calibration and are combined into a unified score that gauges the potential antiviral efficacy of a drug. This integrated approach seeks to boost drug identification confidence, offering a potential solution for detecting therapeutic candidates with pronounced antiviral potency. Once identified a number of compounds were tested for efficacy and toxicity in lung carcinoma cells (Calu-3) infected with SARS-CoV-2. A lead compound was further identified to determine its efficacy and toxicity in K18-hACE2 mice infected with SARS-CoV-2.

**Computational Predictions:** The SNet confidently differentiated between similar and dissimilar drug pairs with an accuracy of 97.28% and AUC of 99.47%. Key compounds identified through these networks included Zinc, Mebendazole, Levomenol, Gefitinib, Niclosamide, and Imatinib. Notably, Mebendazole and Zinc showcased the highest similarity scores, while Imatinib, Levemenol, and Gefitinib also ranked within the top 20, suggesting their significant pharmacological potentials. Further examination of protein binding analysis using explainable AI focused on reverse engineering the causality of the networks. Protein interaction scores for Mebendazole and Imatinib revealed their effects on notable proteins such as CDPK1, VEGF2, ABL1, and several tyrosine protein kinases.

**Laboratory Studies:** This study determined that Mebendazole, Gefitinib, Topotecan and to some extent Carfilzomib showed conventional drug-response curves, with IC50 values near or below that of Remdesivir with excellent confidence all above R2>0.91, and no cytotoxicity at the IC50 concentration in Calu-3 cells. Cyclosporine A showed antiviral activity, but also unconventional drug-response curves and low R2 which are explained by the non-dose dependent toxicity of the compound. Additionally, Niclosamide demonstrated a conventional drug-response curve with high confidence; however, its inherent cytotoxicity may be a confounding element that misrepresents true antiviral efficacy, by reflecting cellular damage rather than a genuine antiviral action. Remdesivir was used as a control compound and was evaluated in parallel with the submitted test article and had conventional drug-response curves validating the overall results of the assay. Mebendazole was identified from the cell studies to have efficacy at non-toxic concentrations and were further evaluated in mice infected with SARS-CoV-2. Mebendazole administered to K18-hACE2 mice infected with SARS-CoV-2, resulted in a 44.2% reduction in lung viral load compared to non-treated placebo control respectively. There were no significant differences in body weight and all clinical chemistry determinations evaluated (i.e., kidney and liver enzymes) between the different treatment groups.

**Conclusion:** This research underscores the potential of repurposing existing compounds for treating COVID-19. Our preliminary findings underscore the therapeutic promise of several compounds, notably Mebendazole, in both *in vitro* and *in vivo* settings against SARS-CoV-2. Several of the drugs explored, especially Mebendazole, are off-label medication; their cost-effectiveness position them as economical therapies against SARS-CoV-2.

## 1 Introduction

The need for cost-effective, safe, and accessible oral therapeutic antivirals for infectious diseases caused by a virus like SARS-CoV-2, responsible for COVID-19, continues to be required particularly in low and middle-income countries (LMICs) (Maxwell et al., 2021; Wang and Yang, 2023). LMICs are particularly challenged in managing COVID-19 due to constrained healthcare resources, notably in the domain of inpatient care. While the global focus has predominantly been on vaccine development, there is an equally critical need for identifying therapeutics with the potential for rapid deployment and ease of administration (Maxwell et al., 2021). This paper introduces a novel computational Artificial Intelligence (AI) platform, named *eVir*, specifically developed for the identification of pharmacological agents with potential efficacy against viral pathogens, including SARS-CoV-2. The core methodology of eVir involves a comparative analysis of the systemic pharmacodynamic profiles of candidate drugs against the known effects of antiviral peptides (AVPs) in the context of the complete human protein-protein interactome. This comprehensive approach assesses how these drugs might interact with and influence the complex network of protein interactions in the human body. AVPs, essentially fragments of human proteins, are instrumental in the innate immune response to viral infections. They act by disrupting vital stages in the viral replication cycle: binding of the virus to the cell surface and its subsequent internalization into endosomal compartments (entry phase), release of the virus from these compartments into the cytosol (fusion phase), and the processing of viral proteins coupled with the replication of the viral genome (replication phase). eVir leverages this biological paradigm to systematically evaluate and rank potential therapeutic agents based on their mechanistic alignment with the antiviral actions of AVPs (Hancock et al., 2016; Agarwal and Gabrani, 2020). eVir is a part of DeepDrug AI, an AI-based drug-discovery pipeline that can create and evaluate novel small molecules as well as repurpose existing ones to rapidly address outbreaks (Naderi et al., 2016; Liu et al., 2017; Pu et al., 2019; Bess et al., 2022).

Each mechanism is critical when considering the effectiveness of antiviral therapy. Entry is critical to inhibit as it reduces the viral load acting on the cell. Similarly, curtailing viral replication is essential to diminish the viral load produced and the subsequent spread to other cells post-infection. Finally, fusion is the process by which a virus can merge directly with the cell membrane, thereby causing infection. Although this process occurs at a rate approximately one-tenth of the standard entry mechanism, it remains a crucial mechanism to target for inhibition (Amidei and Dobrovolny, 2022).

**FIGURE 1:**
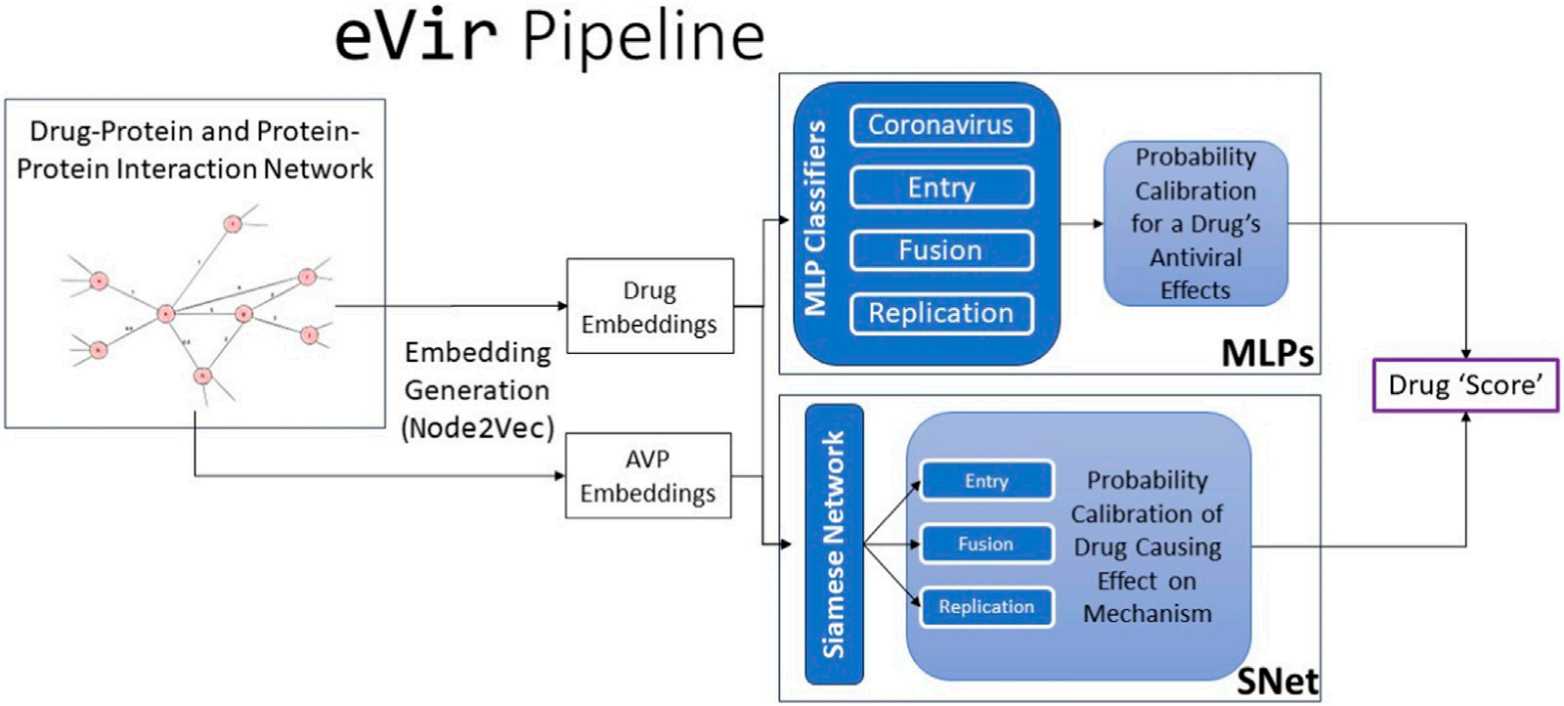
The input to the eVir software pipeline is a combination drug-protein and protein-protein interaction network. We use theNode2Vectool to generate embeddings for the drugs and AVPs. We then use four distinct multi-layer perceptrons (MLPs) to compute probabilities for likelihood of a drug distinctly having an impact on entry, replication, and fusion. We simultaneously use the AVP embeddings and drug embeddings as input into a Siamese Neural Network (SNet) to acquire probabilities of a specific drug effecting each mechanism. Last, we score the input drug by combining MLP and SNet probabilities.

Our eVir pipeline (Figure 1) begins by constructing a human proteome and a corresponding, comprehensive network of the proteins involved in all encompassing processes. We then compute feature vectors for a set of drugs as well as feature vectors in the graph’s same vector space for AVPs. The antiviral peptides (AVPs) utilized in this study were targeted toward these three distinct mechanisms of viral interaction. The most effective peptides were then filtered and used to create three distinct neural networks, each based on the known mechanism of action of a respective peptide. This facilitated identifying pharmaceutical compounds exhibiting specificities aligned with these mechanisms. We then juxtapose these AVP fingerprints with fingerprints of drugs thus identifying drugs which exert a similar (antiviral) effect on the human proteome, akin to the AVPs. *eVir* thus produces a composite score on the predicted impact of a drug on the entry, fusion, and replication of a virus.

eVir is an Artificial Intelligence (AI) application that stands at the intersection of computational biology, systems biology, and pharmaceutical research, embodying a novel paradigm where computational intelligence aids in drug discovery. This project utilizes advanced Deep Learning and Graph Analysis Algorithms, integrating sophisticated neural network architectures such as Siamese Networks and Artificial Neural Networks (Multi-layer Perceptrons), alongside tools for specialized calculating graph embeddings. These algorithms empower eVir to facilitate the identification of novel therapeutic candidates by analyzing intricate biological networks, thereby enhancing the efficiency and efficacy of pharmaceutical research and development. This integration of deep learning with graph analysis places eVir at the forefront of leveraging AI for complex biological data interpretation and drug discovery.

The uniqueness and strength of eVir lie in its versatility. The pipeline, while demonstrated here specifically for SARS-CoV-2 virus interactions, is fundamentally virus agnostic. Its design allows for the seamless integration of new data, making it adaptable to a wide range of viruses. Thus, eVir stands as a forward-looking tool, capable of addressing not only present viral challenges but also preemptively gearing up for potential future pandemics. With every new virus or strain that emerges, the core architecture of eVir remains robust and ready to assist, allowing for timely and effective responses to global health threats.

## 2 Artificial Intelligence software: eVir

### 2.1 Methods

#### 2.1.1 Dataset aggregation and drug-protein network construction

The first step in our process is to construct a network from known protein-protein, known drug-protein interactions, and a selection of AVP datasets. Our network structure was created using the datasets shown in Table 1.

**TABLE 1:** Dataset used for constructing the *eVir* Drug-Protein and Protein-Protein Interaction network.

Dataset	Description of data
AVPdb Qureshi et al. (2014)	2,683 AVPs including 98 from SARS-CoV-1
HIPDB Qureshi et al. (2013)	981 HIV antiviral peptides
hu.map Drew et al. (2021)	17.5 million protein-protein interactions;
CORUM Giurgiu et al. (2019)	4,274 mammalian protein complexes
STRING Snel et al. (2000); Mering et al. (2003); Szklarczyk et al. (2019); Szklarczyk et al. (2023)	4,584,628 proteins from 5,090 organisms;
DrugBank Wishart et al. (2006); Wishart et al. (2008); Wishart et al. (2018)	13,491 drugs
BindingDB Liu et al. (2007); Gilson et al. (2016)	846,857 drugs and 7,605 protein targets

**FIGURE 2:**
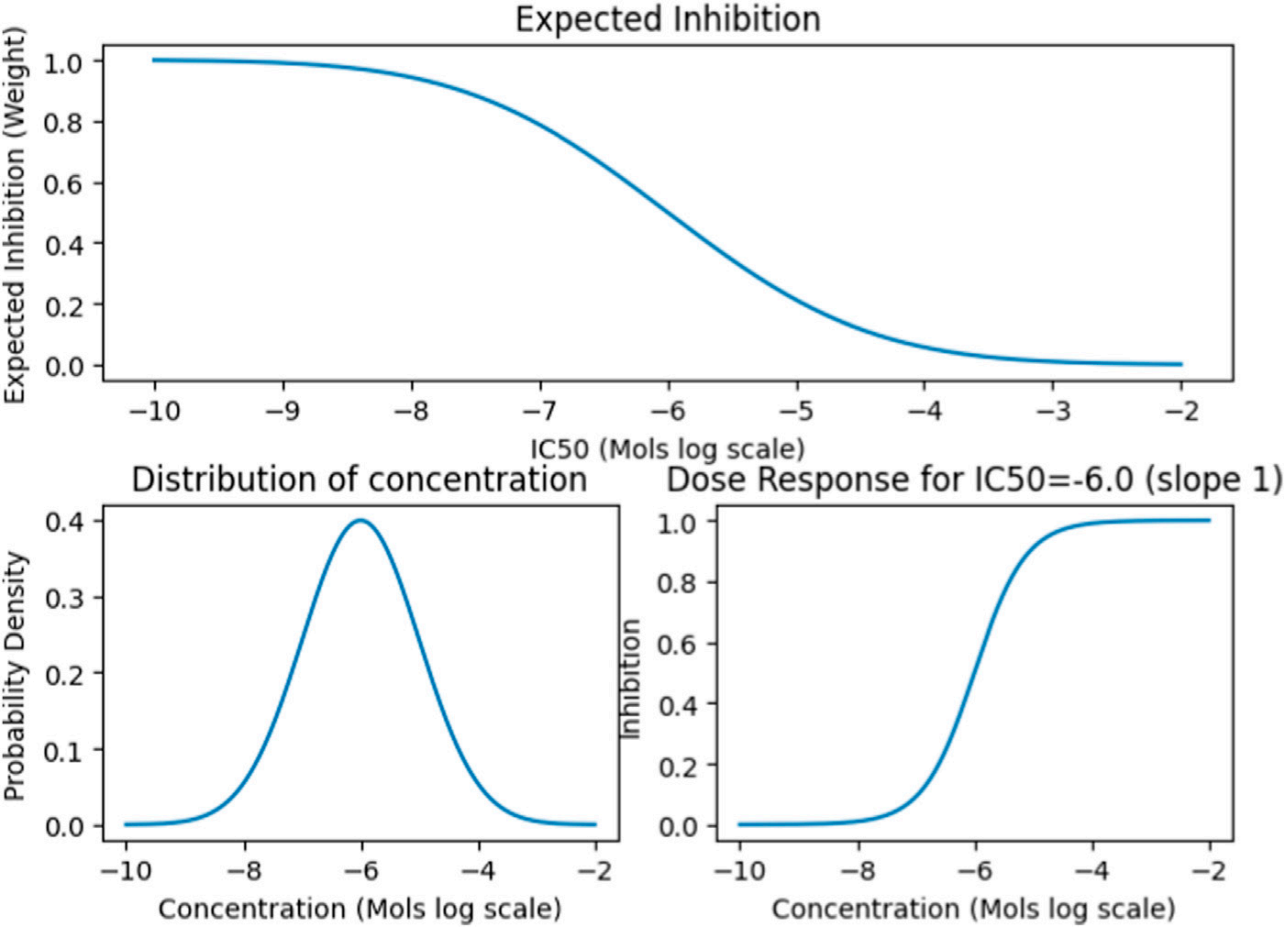
The CDT function simulates the Gaussian distribution of expected drug concentrations, centered around a defined mean with a specific variance. This approach is typically used to model the distribution patterns of a substance’s concentration, assuming it follows a normal distribution. For established drugs, the function utilizes their known dosing and lethal dose (LD50) means and variance to project the expected distribution and influence the outcome. Conversely, for drugs without established parameters, it defaults to using the median concentration value from the entire dataset to estimate the distribution.

We also collected data on the median lethal dose (LD50) and standard human dosage concentrations for approximately 2000 FDA Approved drugs. We then devised a normalization function called the Concentration Distribution Transformation (CDT), to convert the Ki, Kd, EC50, and IC50 values into weights (Figure 2). The CDT was developed by considering both the expected distribution of the drug dosage concentration and a dose-response effect distribution characterized by a sigmoidal curve. Additionally, this approach provided us with a uniform method to integrate results across different drug effect metrics, namely Ki, Kd, EC50, and IC50.

#### 2.1.2 Embedding generation with Node2Vec

Machine learning techniques work over n -dimensional spaces of continuous values. Therefore, one of the most common problems in machine learning is to take an existing space S of potentially non-numeric data and mapping those data to a numeric space. Such a map, π , generates a *latent space* E , or more commonly, an *embedding*: π:S→E . Ideally, E captures as much of the semantics of points in S . It is also desirable for the resulting n -dimensional vectors in E to be dense with a minimum number of dimensions. Hence, in defining π and its resulting space E we have sub-goals in decreasing its sparsity and reducing its dimension.

For *eVir*, we take our space S as a multigraph in which nodes are drugs or proteins and edges are either protein-protein interaction (PPI) edges or drug-protein (DP) edges. We then apply Node2Vec (Grover and Leskovec, 2016) as π to summarize the information in the multigraph. Node2Vec is a graph *embedding* technique based on the Skip-gram architecture (Mikolov et al., 2013a; Mikolov et al., 2013b), which aims to learn a continuous feature representation for nodes in a network. The core idea of Node2Vec is to apply a random walk strategy to generate context for every node, both proteins and drugs. A walk leverages the connections in a graph by capturing topological information around a node by considering different neighborhoods. Each embedding thus encodes insightful feature representations for each node type thus capturing properties of drugs, proteins, and AVPs amongst cellular protein interactions.

As a more detailed description of the Node2Vec embedding construction algorithm, think of our multigraph S as a city map, where every node (drugs and proteins) is a location, and every interaction between nodes is a street connecting these locations. Just as a human may wander through a city, Node2Vec ‘explores’ the network using a random walk: it creates a chain of visited locations by starting at a location, randomly choosing a street to another location, and repeating a fixed number of times. Walks are guided by two parameters that decide whether to revisit a location we have visited before (going in circles) or to venture out into an unexplored territory. The random walks serve as input to a Skip-gram model, a variant of the Word2Vec model, which then predicts the context of a node.

Just as a human would learn much about a city (especially a surrounding neighborhood) through a sequence of random walks starting at the same location, we can distill and learn much about a node, a neighborhood of nodes, and the overall network. For each node, in our multigraph S , this information results in a unique *node embedding* for each drug, protein, and AVP. Each node embedding in E is a vector of continuous values where nodes in S with similar roles or connections in the multigraph have similar embeddings in E . Therefore, the degree of similarity between a drug’s embedding and the embedding of an AVP can indicate the potential effectiveness of a drug: the closer a drug embedding to an AVP embedding, the more likely it is that the drug will similarly inhibit the virus’s mechanism of action.

The Node2Vec algorithm was run with a walk length of 20 steps, reflecting the longest distance between connected protein nodes in the dataset. The algorithm performs 300 such walks for each root node, extensively sampling their local neighborhoods. The return parameter (p) is set to a high value of 20.0, significantly reducing the likelihood of the walks returning to the source node, encouraging exploration away from it. Conversely, the in-out parameter (q) is finely tuned to 2.0, striking a balance between exploring the immediate neighborhood and venturing towards more distant nodes. This setting delicately encourages a breadth-first search strategy for nearby nodes, while still maintaining a general inclination towards extending the walks to farther reaches of the network. Additionally, the walks are weighted, meaning edge weights in the graph will influence the walk trajectories, an important consideration in a network like a protein interaction graph where edge weights can represent the strength or frequency of interactions. This configuration ensures a thorough and balanced exploration of both local and extended graph structures for the analysis of the network structure.

#### 2.1.3 Technological framework: eVir tools and architecture

In the development of eVir, our team employed a sophisticated array of tools and software packages, each selected for its specific strengths in handling the complexities of our algorithms and data processing needs. Central to our architecture is TensorFlow, a robust platform whose advanced functionalities enabled seamless integration and implementation of intricate computational architectures. Complementing TensorFlow, we utilized Pandas and Numpy for their efficiency in data preprocessing, ensuring that our datasets were optimally prepared for analysis. Keras, as an integral part of our toolkit, facilitated streamlined model optimization, allowing for rapid iteration and enhancement of our algorithms.

Further enhancing our capability in network analysis were Gensim and StellarGraph. These packages provided specialized functionalities to augment our capabilities in network analysis and were critical in handling the nuances of network-based data structures and algorithms.

#### 2.1.4 Predicting similarity between drugs and AVPs: Siamese Network

**FIGURE 3:**
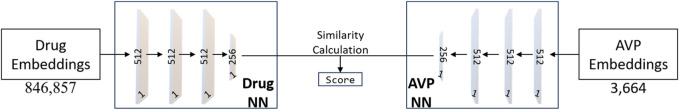
The neural network architecture of the Siamese Network (SNet). The training process utilizes the Adam optimizer with hyperparameters, including a learning rate of 0.00001, epsilon set to 1e-7, and momentum parameters (beta_1 = 0.9, beta_2 = 0.999). Adam’s adaptive learning rate and momentum help accelerate convergence and improve the model’s ability to learn complex patterns from the data.

With input as the set of drug embeddings and AVP embeddings generated by Node2Vec, we predict the effect of an AVP on each mechanism: entry, fusion, and replication. As shown in Figure 3, in a parallel manner, we use drug embeddings also generated by Node2Vec to also predict the effect of drugs on the same mechanisms. Using these two sets of predictions of mechanism effect, we can thus compare a drug embedding for similarity to an AVP embedding thus establishing a measure of similarity and a likelihood for a drug to inhibit as a given AVP. To repurpose a drug, we assess the similarity between drugs and top-performing AVPs, thus pinpointing potential drug candidates exhibiting properties akin to highly efficacious AVPs. As shown in Figure 3, we built a Siamese Network (SNet) (BROMLEY et al., 1993; Koch, 2015) model to predict drug/AVP similarity; we explain our training, tuning, and testing procedures below. To be clear, we constructed three independent Siamese network outputs based on AVP sets, one for each antiviral mechanism (entry, fusion, and replication) from known coronavirus peptides.

*Training*. The SNet was trained using a curated training set consisting of drug pairs. AVP embeddings were selected primarily for their pronounced antiviral potency against particular protein pathways of SARS-CoV-1. In the training of our model, we employed the Tanimoto Coefficient (TC) as the foundation for generating our training datasets. Tanimoto Coefficient is a measure of similarity between two sets and is widely used to compare molecular fingerprints and quantify their similarity. Pairs of drugs manifesting high TC values were integrated as positive instances, whereas those with low TC values were incorporated into the negative dataset. In a novel approach, we leveraged protein pathway information to curate an auxiliary negative dataset. For each protein, the pathways in which it is actively involved were identified. Proteins that did not share these pathways were then paired, resulting in pairs that are presumably functionally dissimilar. These pairs proved invaluable as robust negative instances for our similarity-based sample set.

We also subjected all AVPs to a process commonly known as *masking* that indicates to the model (un)available information when; this enhances the performance of the model. As negative training examples, we chose AVPs with low cosine similarity to ensure diversity and robustness of the model. To optimize the SNet, we employed the *contrastive loss function* during training. This loss function encourages AVPs with similar properties to have *embeddings* close together in the feature space, while pushing dissimilar AVPs further apart. Such an approach aids the model in effectively learning meaningful representations for AVP prediction.

*Testing*. Every model was then evaluated and validated using a distinct test set to assess the model performance with greater unfamiliarity of drug pairs.

We compute the probability of the effectiveness of a particular drug inhibiting entry, fusion, *or* replication using the formula:

$$P_{SNet}\left(x\right)=1-\left(1-P_{SNet:Entry}\left(x\right)\right)\left(1-P_{SNet:Fusion}\left(x\right)\right)\left(1-P_{SNet:Replication}\left(x\right)\right).$$

*Calibration*. We observed a high correlation in prediction scores for entry, fusion, and replication, which led us to take the mean of these three distances before calibration. To calibrate output from the SNet, the positive set was drugs targeting cyclin-dependent kinase. This class of drugs has been proposed as possible treatments for COVID-19 (Gargouri et al., 2021). This hypothesis was supported by the SNet consistently favoring scores for these drug classes.

#### 2.1.5 Predictive differentiation between drugs and AVPs: MLP classification

For each antiviral mechanism, we architected, trained, and tested a multilayer perceptron (MLP): a straightforward form of neural network. Our goal for the set of MLPs is to predict whether a drug can be classified as having entry, fusion, and replication proximal to AVPs. We trained each MLP model using SARS-CoV-1 AVPs as positive examples and a comprehensive dataset comprising 800,000 pharmaceutical compounds as negative examples. The input data consisted of drug and AVP embeddings. Given the imbalance in the number of drugs compared to AVPs, each drug was assigned a minimal weight to maintain balance within the training set.

**FIGURE 4:**
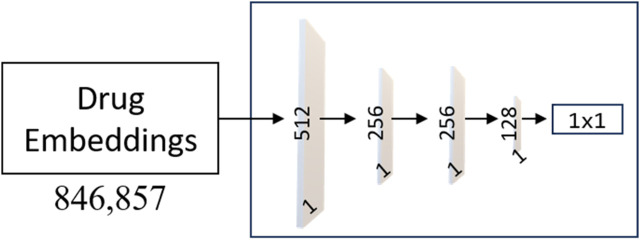
Architecture of the MLP Networks. The model consists of an input layer with 512 neurons, followed by two hidden layers with 256 and 128 neurons, respectively. Each hidden layer utilizes the Rectified Linear Unit (ReLU) activation function, promoting non-linearity and feature extraction. To enhance the model’s learning ability, weight masking is incorporated to selectively emphasize important connections and reduce noise.

Our underlying hypothesis for the MLPs is that a drug exhibiting dissimilarity to AVPs yields an extremely low output value (close to 0). In contrast, a drug that cannot be differentiated from AVPs would produce output values approaching 1. The training process for each MLP (architecture presented in Figure 4) employed the Adam optimizer with hyperparameters, including: a learning rate of 0.00001 and momentum parameters (beta_1 = 0.9, beta_2 = 0.999) for optimization. The model is trained using cross-entropy loss, which is a prevalent and well-established choice for binary classification tasks. The performance is averaged over 10 independent runs of the training process to ensure robustness and reliability.

As shown in Figure 4, the output layer of each MLP contains a single neuron with a sigmoid activation function, producing a score for drug classification. L2 regularization is applied to the input and hidden layers with a coefficient of 0.03 to prevent overfitting, and dropout layers with a rate of 0.5 is used in the input and first hidden layer to enhance generalization. The proposed model shows promising results and presents a potential solution for accurate and practical drug classification tasks involving AVPs.

We compute the probability of the effectiveness of a particular drug using the formula in which x is a given drug:

$$P_{MLP}\left(x\right)=P_{MLP:Corona}\left(x\right)\cdot \left(1-\left(1-P_{MLP:Entry}\left(x\right)\right)\left(1-P_{MLP:Fusion}\left(x\right)\right)\left(1-P_{MLP:Replication}\left(x\right)\right)\right).$$

This formula attempts to compute a single probability for the MLP subsystem by determining whether a given drug acts on Coronavirus by having high scores for inhibiting entry, fusion, and replication.

*Calibration.* For the MLP calibration, we used drugs that directly target SARS-CoV-1 as our positive set.

#### 2.1.6 Transforming a score into a probability

To systematically consolidate the predictions generated by both the SNet and the MLPs into a unified score, we used a Platt probability calibration (Niculescu-Mizil and Caruana, 2005a; Niculescu-Mizil and Caruana, 2005b): using positive and negative datasets, we used one-dimensional logistic regression to fit a sigmoid function to the scores. As the sigmoid function is a monotonically increasing function, this calibration function alters the scale of the scores, while leaving the relative ranking among different unaltered. The negative set was constructed of drugs lacking common pathways with known antivirals or those drugs with a TC less than 0.2 to known antiviral drugs.

#### 2.1.7 The final drug score

After calibration of MLP prediction scores and SNet prediction scores, a final probability score is calculated assuming statistical independence:

$P_{final}\left(x\right)=P_{MLP}\left(x\right)+P_{SNet}\left(x\right)-P_{MLP}\left(x\right)\cdot P_{SNet}\left(x\right)$ where x is a given drug.

This method inherently assumes that these probabilities are independent variables, meaning the occurrence of one event does not affect the probability of the others. This is a standard assumption in probabilistic models, especially when variables are derived from different data sets or represent distinct biological processes.

If either the MLP or the SNet model accords a drug with a favorable score, it is considered adequate for potential identification. If both models yield positive predictions (e.g., values close to 1), the final score of a drug is also close to 1. In such a case, where both models assign high probabilities, it indicates a heightened confidence level in the classification of a drug.

By applying this statistical independence assumption and considering the contributions of both models, we aim to increase the robustness and interpretability of the final drug classification process, allowing for more confident identification of potential candidates.

### 2.2 eVir results

#### 2.2.1 SNet

**FIGURE 5:**
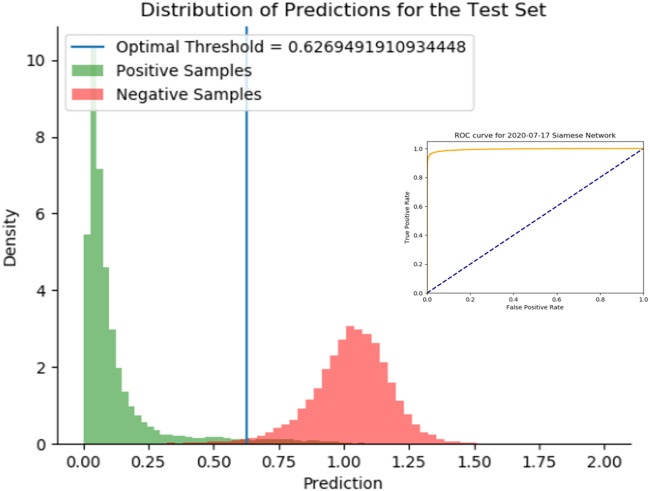
Histogram of predictions from the SNet on a test set of drug-drug pairs. Green peaks signify similar drug pairs and the red peaks display the drug pairs that are dissimilar. The optimal threshold is based on a J-statistic to find the best separation between the two populations. Embedded figure: a Receiver Operating Characteristic (ROC) curve verification of the SNet performance (97.28% Accuracy, 99.47% AUC). We have high confidence with known data that SNet can discriminate between similar and dissimilar drug pairs.

We demonstrate the effectiveness of the SNet by considering a test set of drug-pairs as shown in Figure 5. Our test set for the SNet contains known similar and non-similar drug pairs used for training the model. Thus, an effective model will result in a strong bimodal distribution in which one mode represents known, similar drug-drug pairs and the other mode represents known, dissimilar drug-drug pairs. We observe this strong bimodal distribution in Figure 5 over 15,000 similar drug-drug pairs and an equal number of dissimilar drug-drug pairs. The inserted ROC curve in Figure 5 having an Area under the ROC Curve (AUC) of 0.9947 is strong statistical evidence that the SNet acts reliably as a discriminator model.

We present in Table 2 a list of the top 30 ranked, FDA-approved compounds with the corresponding Euclidean distances (computed as the square root of the sum of squared differences between individual dimensions of the two points) for each viral mechanism. We calculated the distance between each drug and each AVP for each mechanism of action. The distances were averaged over a selection of pharmacology-informed optimal AVPs for each mechanism of action. Therefore, smaller values of the Euclidean distance in Table 2 suggest that a drug’s effect closely mirrors that of an AVP for a specific viral mechanism.

**TABLE 2:** SNet distance scores for a list of the top 30 ranked, FDA-approved compounds along with their corresponding Euclidean distance obtained from the entry, fusion and replication clusters. The Euclidean distance ranges from 0 (highest possible score) to 2 (lowest possible score), indicating the similarity between the compounds and AVPs corresponding to entry, fusion, and replication based on their pharmacological activities and therapeutic potential.

DrugBank ID	Name	Entry (SNet)	Fusion (SNet)	Replication (SNet)
DB01593	Zinc	0.0835	0.1365	0.1151
DB06626	Axitinib	0.1056	0.1258	0.1531
DB00465	Ketorolac	0.106	0.1088	0.1894
DB00643	Mebendazole	0.1072	0.0796	0.2033
DB08865	Crizotinib	0.1078	0.1098	0.1877
DB05109	Trabectedin	0.1154	0.0812	0.2136
DB06616	Bosutinib	0.1159	0.0733	0.2182
DB05294	Vandetanib	0.1269	0.0842	0.2267
DB11828	Neratinib	0.1285	0.1154	0.2061
DB00558	Zanamivir	0.1297	0.1658	0.1782
DB06616	Bosutinib	0.1327	0.0864	0.2305
DB13153	Levomenol	0.1329	0.1467	0.2079
DB06595	Midostaurin	0.1331	0.0877	0.2298
DB00742	Mannitol	0.134	0.1128	0.2234
DB00317	Gefitinib	0.1351	0.0916	0.2311
DB01259	Lapatinib	0.1362	0.1151	0.2225
DB12001	Abemaciclib	0.1376	0.1827	0.1292
DB09330	Osimertinib	0.1382	0.0837	0.2432
DB00864	Tacrolimus	0.1387	0.1033	0.2461
DB06803	Niclosamide	0.1395	0.1061	0.2244
DB00619	Imatinib	0.1455	0.0955	0.2424
DB08912	Dabrafenib	0.1467	0.0821	0.249
DB09073	Palbociclib	0.1469	0.1316	0.2082
DB08865	Crizotinib	0.1491	0.1029	0.2419
DB11689	Selumetinib	0.1493	0.1026	0.2426
DB11986	Entrectinib	0.1507	0.1133	0.2366
DB00398	Sorafenib	0.1556	0.1017	0.2525
DB00864	Tacrolimus	0.1575	0.1263	0.2618
DB00530	Erlotinib	0.1584	0.1066	0.2557

We describe some of the pharmaceutical compounds that received notable scores from the SNet for entry, fusion, and replication:

• Zinc, an essential mineral that plays a vital role in various cellular functions, including immune support and enzymatic reactions;
• Mebendazole, an anthelmintic agent commonly used to treat parasitic worm infections;
• Levomenol, a natural compound (nutraceutical) found in chamomile with known anti-inflammatory and antimicrobial properties;
• Gefitinib, a tyrosine kinase inhibitor commonly used in the treatment of non-small cell lung cancer by targeting specific proteins responsible for cancer growth and progression;
• Niclosamide, an antiparasitic drug with potential anti-cancer properties currently being investigated for its role in inhibiting tumor growth and metastasis; and
• Imatinib, one of the first discovered tyrosine kinase inhibitors that revolutionized the treatment of chronic myeloid leukemia.

Each of these compounds have shown promising pharmacological properties in their respective therapeutic areas, making each a significant candidate for further research and clinical application. Mebendazole and Zinc obtained the highest similarity scores, suggesting their potential as strong candidates for therapeutic interventions due to their pharmacological activities. Imatinib, Levemenol, and Gefitinib also scored favorably, ranking within the top 20, indicating significant pharmacological activities in their respective therapeutic areas. Levemenol and Zinc are particularly interesting as potential therapies, given their accessibility as nutraceuticals.

#### 2.2.2 MLP network results

The MLPs module consists of 4 individual MLP models, one for Coronavirus AVPs and one for each viral mechanism: entry, fusion, and replication. Table 3 presents a list of the top 30 FDA-approved compounds ranked according to the score from the Coronavirus MLP. In contrast to the SNet, MLPs output a probability, therefore, a score near 1 indicates a strong likelihood that the drug is similar acting to the set of AVPs used to train the network.

**TABLE 3:** MLP classification results for a list of FDA approved drugs alongside their corresponding probability scores obtained from Entry, Fusion, Replication and Coronavirus predictive networks. The classification probability score ranges from 0 (lowest possible score) to 1 (highest possible score), indicating the probability that a particular drug’s pharmacological activities and therapeutic potential is classified as a compound in the AVP set used to train the corresponding MLP.

DrugBank ID	Name	Corona (MLP)	Entry (MLP)	Replication (MLP)	Fusion (MLP)
DB00558	Zanamivir	0.9094	0.813	0.4871	0.8796
DB04348	Taurocholic acid	0.8842	0.7994	0.4509	0.7902
DB00091	Cyclosporine	0.848	0.9071	0.2459	0.1886
DB00558	Zanamivir	0.8447	0.8762	0.6867	0.8223
DB09570	Ixazomib	0.843	0.87	0.8225	0.1155
DB08912	Dabrafenib	0.8273	0.1521	0.0753	0.9719
DB04835	Maraviroc	0.8073	0.9698	0.1608	0.0549
DB00091	Cyclosporine	0.7703	0.8646	0.5327	0.4575
DB00091	Cyclosporine	0.7694	0.7884	0.2206	0.1842
DB00091	Cyclosporine	0.7639	0.8271	0.1952	0.1424
DB06616	Bosutinib	0.7619	0.4362	0.1013	0.6597
DB00091	Cyclosporine	0.7587	0.8456	0.5234	0.4309
DB08889	Carfilzomib	0.754	0.6684	0.4825	0.2293
DB12001	Abemaciclib	0.7438	0.8836	0.2734	0.0157
DB08865	Crizotinib	0.7076	0.5645	0.0314	0.1091
DB06809	Plerixafor	0.6486	0.9087	0.0598	0.0366
DB06614	Peramivir	0.646	0.2581	0.1409	0.294
DB00643	Mebendazole	0.6323	0.3207	0.0847	0.3885
DB00558	Zanamivir	0.625	0.294	0.1492	0.378
DB13153	Levomenol	0.6191	0.1641	0.093	0.7499
DB00091	Cyclosporine	0.6132	0.6568	0.0518	0.1148
DB00369	Cidofovir	0.6037	0.2798	0.1857	0.8517
DB15102	Pemigatinib	0.5876	0.142	0.0433	0.6876
DB00722	Lisinopril	0.5759	0.2991	0.2068	0.7406
DB00465	Ketorolac	0.5698	0.1284	0.4934	0.3201
DB00619	Imatinib	0.5696	0.0892	0.0092	0.3522
DB06595	Midostaurin	0.563	0.251	0.0751	0.1797
DB09054	Idelalisib	0.5425	0.1665	0.0496	0.485
DB09073	Palbociclib	0.5362	0.2878	0.0881	0.1416
DB01301	Rolitetracycline	0.523	0.2362	0.6407	0.1007

Among the pharmaceutical compounds that received high scores from the MLP networks were Ixazomib, a proteasome inhibitor; vitamin D; kinase inhibitors Dabrafenib and Bosutinib; Cyclosporine, an immunosuppressant; and Levomenol, a compound found in chamomile known for its anti-inflammatory and antimicrobial properties (Wishart et al., 2006; Wishart et al., 2008; Wishart et al., 2018). The consistently high scores observed for the proteasome inhibitors and tyrosine kinase drug classes suggest that these two classes of drugs might involve important pathways considered significant by the predictive network. As a result, they were passed to a team of pharmacologists for further consideration.

*Protein Pathway Causality Analysis.* We conducted analyses on each individual MLP model itself, focusing on the resulting ranking of drugs. Our objective was to reverse engineer the causality of the networks with the hopes of explaining some of the decisions of the networks. We used known interactions of the drugs that received high rankings to identify important proteins that each network appeared to favor. Our goal was to gain insights into the underlying mechanisms and pathways that contributed to a network’s predictions and to uncover potential targets and pathways associated with the pharmacological activities of each highly ranked drug.

For Mebendazole, protein interaction scores for 22 proteins were calculated based on *p*-values for statistical significance and are presented in Table 4: The top four proteins with the most significant interaction scores were calmodulin-domain protein kinase 1 (CDPK1) from the parasite *Toxoplasma gondii* (a score of 0.67), vascular endothelial growth factor receptor 2 (VEGF2) (0.62), Abelson tyrosine protein kinase 1 (ABL1) (0.57), and proto-oncogene tyrosine protein kinase Src (SRC) (0.55).

**TABLE 4:** A list of proteins (in)directly affected by Mebendazole with corresponding protein interaction scores derived from a causality analysis. Organism types and UniProt ID are also provided. A higher score (green) indicates a larger effect of Mebendazole on the protein.

Protein Interaction Score for Mebendazole	Protein Name	Organism	UniProt ID
**0.67**	Calmodulin-domain protein kinase 1	*Toxoplasma gondii*	Q9BJF5
**0.62**	Vascular endothelial growth factor receptor 2	*Human*	P35968
**0.57**	Abelson tyrosine-protein kinase 1	*Human*	P00519
**0.55**	Proto-oncogene tyrosine-protein kinase Src	*Human*	P12931
0.43	Integrin alpha-5	*Human*	P06756
0.4	Adenylate cyclase type 6	*Human*	O43306
0.2	Genome polyprotein	*West Nile Virus*	P06935
0.16	Lysosomal-associated membrane protein 3	*Human*	P08962
0.15	Trans-activator protein BZLF1	*Epstein-Barr Virus*	P03206
0.15	RNA-directed RNA polymerase subunit P3	*Influenza A Virus*	P03428
0.11	Capsid protein	*Hepatitis B Virus*	Q76R61
0.09	Integrin alpha-3	*Human*	P26006
0.09	Tyrosine-protein kinase Yes	*Human*	P07947
0.08	Protein LANA1	*Herpes Virus*	Q9QR71
0.08	Signal peptide peptidase-like 2A	*Human*	Q8TCT8
0.07	Tyrosine-protein kinase Lyn	*Human*	P07948
0.02	Tyrosine-protein kinase Fyn	*Human*	P06241
0.02	Insulin-like growth factor 1 receptor	*Human*	P08069
0.02	Protein kinase C alpha type	*Human*	P17252
0.02	Insulin receptor	*Human*	P06213
0.02	Ephrin type-A receptor 2	*Human*	P29317
0.01	Casein kinase I isoform gamma-3	*Human*	Q9Y6M4

In Table 5, a similar analysis for Imatinib identified 36 proteins, including several tyrosine protein kinases.

**TABLE 5:** A list of proteins (in)directly affected by Imatinib and the corresponding protein interaction scores derived from a causality analysis. Organism types and UniProt ID are also provided. A higher score (green) indicates a larger effect of Imatinib on the protein.

Protein interaction score for imatinib	Protein name	Organism	UniProt ID
**0.83**	Serine/threonine-protein kinase Chk1	*Human*	O14757
**0.83**	Protein kinase C eta type (PKC-L)	*Human*	P24723
**0.83**	Myotonin-protein kinase	*Human*	Q09013
**0.83**	cGMP-dependent protein kinase 1	*Human*	Q13976
**0.83**	Aurora kinase B	*Human*	Q96GD4
**0.83**	Serine/threonine-protein kinase D2	*Human*	Q9BZL6
**0.83**	Interleukin-1 receptor-associated kinase 3	*Human*	Q9Y616
**0.8**	Platelet-derived growth factor receptor beta	*Human*	P09619
**0.8**	Discoidin domain-containing receptor 2	*Human*	Q16832
**0.79**	Platelet-derived growth factor receptor alpha	*Human*	P16234
**0.76**	Breakpoint cluster region protein	*Human*	P11274
**0.75**	Phosphatidylinositol 5-phosphate 4-kinase type-2 gamma	*Human*	Q8TBX8
**0.75**	Tyrosine-protein kinase Blk	*Human*	P51451
**0.73**	Homeodomain-interacting protein kinase 4	*Human*	Q8NE63
**0.73**	Cyclin-G-associated kinase	*Human*	O14976
**0.73**	Interleukin-1 receptor-associated kinase 1	*Human*	P51617
**0.72**	Ephrin type-A receptor 8	*Human*	P29322
**0.72**	Tyrosine-protein kinase FRK	*Human*	P42685
**0.72**	Platelet-derived growth factor receptor beta	*Rat*	Q05030
**0.72**	RAF proto-oncogene serine/threonine-protein kinase	*Human*	P04049
**0.72**	Maternal embryonic leucine zipper kinase	*Human*	Q14680
**0.71**	Dual specificity protein kinase CLK4	*Human*	Q9HAZ1
**0.71**	Tyrosine-protein kinase Fgr	*Human*	P09769
**0.71**	Mitogen-activated protein kinase kinase kinase 20	*Human*	Q9NYL2
**0.7**	Tyrosine-protein kinase Fyn	*Human*	P06241
**0.7**	Mitogen-activated protein kinase 10	*Human*	P53779
**0.7**	Serine/threonine-protein kinase B-raf	*Human*	P15056
**0.7**	Serine/threonine-protein kinase TNNI3K	*Human*	Q59H18
**0.7**	Dual specificity protein kinase CLK1	*Human*	P49759
**0.69**	Mitogen-activated protein kinase 8	*Human*	P45983
**0.69**	Serine/threonine-protein kinase 17A	*Human*	Q9UEE5
**0.69**	Serine/threonine-protein kinase PLK4	*Human*	O00444
0.64	Inner centromere protein	*Human*	Q9NQS7
**0.61**	Vascular endothelial growth factor receptor 2	*Human*	P35968
**0.61**	Abelson tyrosine-protein kinase 1	*Human*	P00519
**0.61**	Proto-oncogene tyrosine-protein kinase Src	*Human*	P12931

#### 2.2.3 The eVir generated list of recommended oral therapeutics

Table 6 presents our list of the recommended oral therapies from eVir.

**TABLE 6:** This table presents the prioritized list of drug therapies as determined by aggregate AI evaluations across multiple models. The “Score” column represents the comprehensive AI-derived rating, “SNet” denotes a weighted mean derived from the scores of the Siamese networks, and “Corona MLP” pertains to the ratings generated by the MLP model specifically trained for coronaviruses. Chemical structures of these compounds can be found at https://www.chemspider.com/.

Name	IITRI EC50	IC50 from the literature	Score	SNet	Corona (MLP)
Cholecalciferol	22.3 mM	3–10 μM Pickard et al. (2021)	0.9	0.03	0.89
Bosutinib			0.92	0.56	0.82
Isoxsuprine	1095 nM		0.86	0.53	0.7
Levomenol	∼1651 nM 0.35 μM (VIDO)		0.82	0.51	0.64
Mebendazole	48.55 nM 102 nM		0.82	0.57	0.58
Ketorolac			0.81	0.56	0.56
Imatinib	∼6.310 nM	9.8–17.6 μM Weston et al. (2020)	0.75	0.52	0.48
Niclosamide	982.5 nM	0.28 μM Gassen et al. (2021)	0.72	0.52	0.41
Gefitinib	62 nM 44 nM		0.58	0.53	0.1
Tacrolimus	∼807.8 nM	5.4 μM Dittmar et al. (2021)	0.47	0.43	0.06
Zinc	959.9 nM		0.13	0.6	0.21
Cyclospirine A	∼5 μM 2.1 μM	16 μM Dittmar et al. (2021)	0.88	0.34	0.82
Topotecan	9.362 nM 35 nM		0.19	0.08	0.12
Carfilzomib	5.741 nM 27 nM		0.84	0.27	0.81

## 3 Cell studies

### 3.1 Methods

eVir software identified several potential antiviral drugs through analysis of host cell–SARS CoV-2 virus, protein-protein interactions. The validation of therapeutics recommended by eVir was performed *in vitro* using human lung cancer (Calu-3) cells seeded into 96 well plates, at 40,000 cells/well. The concentration where drug toxicity occurred was determined using MTT assay. For the infection assays cells were inoculated with SARS-CoV-2 WA-1 strain at a 0.01 multiplicity of infection (MOI) and incubated for 60–90 min. The cells were provided with EMEM with 2% FBS and test compounds and incubated for an additional 48 h. Absorbance readings for each well were collected by Softmax Pro software and imported into a Microsoft Excel spreadsheet. Outliers were detected by Grubbs’ test in the built-in analysis of Graphpad Prism 9.

A. Experimental Methods: Efficacy and cytotoxicity were determined for each test article (TA) and control compound at eight concentrations in Calu3 cells, in triplicate.
  1. Cytotoxicity: Calu-3 cells were seeded at 40,000 cells/well in separate 96-well plates 48 ± 2 h prior to the day of assay, and half of the media was replaced at 24 ± 1 h. At the study’s initiation, cells exhibited over 90% confluency. Diluted TA was incubated with the cells in a humidified chamber at 37°C ± 2°C in 5% ± 2% CO2. Post 48 ± 2 h of incubation, cellular metabolic activity was measured using MTT (3-(4,5-dimethylthiazol-2-yl)-2,5-diphenyl tetrazolium bromide) assay, indicative of cell viability, proliferation, and cytotoxicity. Absorbance was determined at 540 nm using a microplate reader.
  2. Efficacy of viral inhibition. For efficacy testing, Calu-3 cells were prepared similarly as in the cytotoxicity assessment. Post seeding, cells were inoculated with SARS-CoV-2 at a multiplicity of infection (MOI) of 0.01 TCID50/cell and incubated for 60–90 min. Immediately following incubation with SARS-CoV-2, virus inoculum was removed, cells were washed twice with warmed PBS, and the appropriate wells were overlaid with 1x EMEM supplemented with 2% FBS pre-warmed to 37°C containing test compounds at the concentrations specified in the section II-C. Plates were then incubated in a humidified chamber at 37°C ± 2°C in 5% ± 2% CO2. After 48 ± 2 h post infection, cells were fixed and evaluated for the presence of virus using an immunostaining assay (see below).
  3. Controls: The virus control (VC) wells contained only SARS-CoV-2 and Calu-3 cells and acted as the infected control. The cell control (CC) wells contained cells only, no virus, and served as the background control. VC (n = 12) and CC (n = 12) were loaded in each 96 well-plate.
B. Immunostaining Assay: Cells were fixed with 80% cold acetone and blotted with anti-coronavirus nucleoprotein (NP) monoclonal antibodies (EastCoast Bio Cat No. HM1056 and HM1057), followed by peroxidase-conjugated goat anti-mouse IgG (Fitzgerald, Cat No. C21010801, 1:1500). Wells were then developed using ABTS Peroxidase Substrate System (SeraCare, Cat No. 10531349). The development was stopped, and the plates were read at 405 nm with a 490 nm reference using an ELISA plate reader (Molecular Devices SpectraMax M2).
C. Data Analysis: Absorbance readings for each well were collected by Softmax Pro software (version 7.0.3 GXP; San Jose, CA) and imported into a Microsoft Excel spreadsheet for further calculations. Outliers were detected by Grubbs’ test in the built-in analysis of Graphpad Prism 9. For each well, virus inhibition was quantified by calculating the percentage reduction in absorbance (measured at 405 nm) relative to both the mean absorbance of the virus control wells and the mean absorbance of the cell control wells on the same plate. This was achieved using the following formula:

$$Percentage\;Virus\;Reduction=100-\left[\left(\frac{Well\;A405-Mean\;Cell\;Control\;A405}{Mean\;Virus\;Control\;A405-Mean\;Cell\;Control\;A405}\right)\times 100\right]$$

The data was plotted using Graphpad Prism 9 and concentration-response curves and inhibition concentrations (IC50’s) for each test article was calculated by 4-parameter non-linear regression curve fitting.

$$IC50=a+\left(\frac{d-a}{1+{\left(\frac{x}{c}\right)}^{b}}\right)$$

Where: a = upper asymptote b = slope at inflection point, c = inflection point, d = lower asymptote (g = asymmetry factor could be also included).

The IC50 is defined as the calculated reciprocal of the log10 dilution resulting in a 50% reduction of the absorbance value of the virus control wells (50% A405 reduction), indicating a 50% reduction in viral activity. The Selectivity index (SI) was calculated to evaluate the safety margin of the TA. It is defined as the ratio of the TA’s CC50 to the TA’s IC50 values (SI = CC50/IC50z).

### 3.2 Results

**FIGURE 6:**
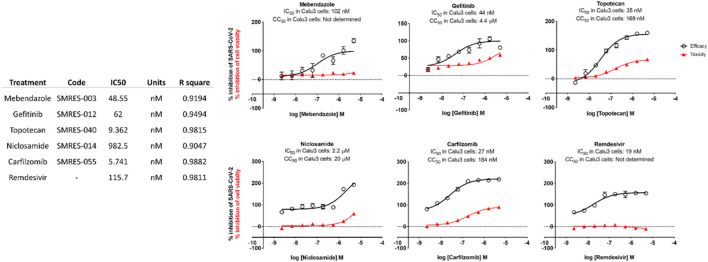
*In vitro* IC-50 drug-response curves for Calu-3 cells.

Test compounds were evaluated against SARS-CoV-2 in Calu-3 cells. The cellular toxicity (CC50) and antiviral efficacy (IC50) data are summarized in Figure 6: *In vitro* IC-50 drug-response curves for Calu-3 cells. Mebendazole, gefitinib, topotecan, and, to a lesser extent, carfilzomib showed conventional drug-response curves, with IC50 values near or below that of remdesivir with excellent confidence that were all above R2 > 0.91 with no cytotoxicity at the IC50 concentration (Figure 6). Cyclosporine A showed antiviral activity, but also unconventional drug-response curves and low R2, which are explained by the dose-independent toxicity of the compound. Additionally, Niclosamide demonstrated a conventional drug-response curve with high confidence; however, its inherent cytotoxicity may be a confounding element that misrepresents true antiviral efficacy, by reflecting cellular damage rather than a genuine antiviral action. Remdesivir was used as a control compound and was evaluated in parallel with the submitted test articles and had conventional drug-response curves validating the overall results of the assay.

Taken together, these preliminary findings indicate that several novel compounds exhibit noteworthy *in vitro* activity against SARS-CoV-2. Specifically, Mebendazole, Gefitinib, Topotecan, Niclosamide, and Carfilzomib stand out for their pronounced antiviral activity with minimal to no apparent cytotoxic effects, as indicated by their lack of influence on cell viability. The discovery of these repurposed compounds’ efficacy against SARS-CoV-2 establishes a foundation for further exploration into their potential therapeutic applications in the context of SARS-CoV-2 infections.

## 4 Preliminary animal study

### 4.1 Methods

Experimental Design: Six hours following challenge with 5 × 103 TCID50 SARS-CoV-2, human transgenic female K18-hACE2 (B6.Cg-Tg(K18-ACE2)2PrlmnJ) mice were administered placebo, Mebendazole at 50 mg/kg po once a day for 6 consecutive. Following the treatment period, the mice were humanely euthanized, and the viral load in their lungs was quantified using an RT-qPCR assay. For the RT-qPCR assay, RNA was extracted from lung samples preserved in RNA/DNA Shield using the Quick-RNA Viral Kit (Zymo Research), adhering to the manufacturer’s instructions. The RT-qPCR cycling conditions were as follows: 50°C for 15 min (RT), then 95 °C for 2 min (denature), then 40 cycles of 10s at 95°C, 45s at 62°C. Primers used for SARS-CoV-2 detection: 2019-nCoV_N1-F 5′-GAC​CCC​AAA​ATC​AGC​GAA​AT-3′ 2019-nCoV_N1-R 5′-TCT​GGT​TAC​TGC​CAG​TTG​AAT​CTG -3′ Probe: 2019-nCoV_N1-P: 5′-FAM-ACCCCGCATTACGTTTGGTGGACC-BHQ1-3’. Additional Experimental endpoints were also determined including body weight and body weight change and clinical chemistry determinations such as BUN, AST, and ALT.

Challenge Virus Administration: Prior to challenge, the mice were anesthetized using an injection of a ketamine (100 mg/kg)/xylazine (10 mg/kg) mixture. Once anesthetized, each mouse was held with its nose pointing upward, and 0.030 mL of challenge material was delivered dropwise into the nares. Mice were held upright to allow the challenge virus solution to be inhaled thoroughly before being returned to their cage. The challenge dose was 5 × 103 TCID50 SARS-CoV-2 per animal.

Dose Administration: Placebo or test material dosing formulations were administered once daily by oral gavage to each surviving mouse on Study Days 0–10. On Study Day 0, doses were administered 4–6 h after challenge. On Study Days 1–10, doses were administered 24 ± 2 h after the preceding dose.

Mortality and Moribundity: During the quarantine period, all mice were observed at least once daily for survival. Following challenge (on Study Day 0) and throughout the remainder of the study, all surviving mice were observed twice daily for mortality or evidence of moribundity. Any abnormal clinical signs were recorded in ToxData^®^. The Study Director (or designee) was notified of any severely ill or moribund animal prior to euthanasia. Animals meeting a previously determined definition of moribundity in accordance with accepted animal care practices (ACUP) and relevant standard operating procedures (SOPs) were euthanized within 6 hours of this determination and counted as dead for the study.

Body Weights: All surviving mice were individually weighed at the following time points:

• Within 2 days of receipt (not reported)
• Prior to randomization (not reported)
• Prior to challenge on Day 0
• Once daily on Study Days 114

The percent change in body weight from the Day 0 body weight was calculated for each mouse.

Euthanasia and Necropsy: On Study Day 6 (77/group) or Study Day 14 (all surviving) mice were euthanized via an intraperitoneal (IP) injection of Beuthanasia-D solution (0.67 mL/kg; Pentobarbital/Phenytoin solution) and exsanguinated. Death was confirmed by the absence of an observable heartbeat and respiration for a period of 3–5 min.

• In the IITRI ABSL-3 suite, a gross necropsy examination was performed on the lungs of each sacrificed mouse on Study Day 6 and 14. The necropsy for viral titers was conducted on Study Day 6. The left lung was harvested from 7 study animals per treatment group (if available) for viral titers. Tissues for both TCID50 and RTqPCR viral titers were weighed and flash frozen in liquid nitrogen. Samples were stored at ≤-65°C until analyzed.
• The following organs were also collected from each sacrificed mouse on Study Day 6 and 14 and fixed in 10% neutral buffered formalin (NBF) for microscopic examination.
• Nasal turbinate
• GI tract (stomach, jejunum, ileum, and colon)
• Leg bone
• Bronchial lymph nodes
• Right lung
• The right lung of each sacrificed mouse was removed, inflated by intratracheal infusion of 10% NBF, and then immersed in 10% NBF for fixation. Fixed tissues were sent to Charles River Laboratories, Pathology Associates (PAI) for histopathological evaluation.
• Animals that were found dead, died accidently or were determined to be moribund were euthanized with beuthanasia (if necessary) and removed from the study without further processing.

Clinical Chemistry: On Study Day 6 and Study Day 14, blood was collected from the retro-orbital sinus of each mouse for clinical chemistry determinations during euthanasia. Blood was collected into tubes, allowed to clot, and centrifuged to obtain serum. Liver and kidney enzymes AST, ALT and BUN were evaluated using a Beckman Coulter AU480 Clinical System (Beckman Coulter, Inc.; Brea, CA).

Histopathology: All tissues and organs listed above were evaluated microscopically by a board-certified veterinary pathologist. Tissues to be examined microscopically were trimmed, processed routinely, embedded in paraffin and stained with hematoxylin and eosin.

TCID50 Analysis of Lung Tissue: Vero C1008 (E6) cell monolayers were observed for ≥80% confluency using an inverted microscope. Lung tissue sample was quickly thawed in a 37°C ± 2°C water bath and immediately homogenized in 1x PBS and used for assay. Dilutions of the homogenized samples were prepared with infection media [e.g. DMEM-2: Dulbecco’s Modified Eagle’s Medium (DMEM) + 2% Heat Inactivated Fetal Bovine Serum (FBS), 1% Penicillin/Streptomycin] in sterile 96 deep wells (typically tested from 10 to 1 to 10-8 or 10-1 to 10-11) of each sample by making a 10-fold dilution series (e.g. add 60 µL of sample to 540 µL of infection media). Dilution schemes were adjusted when appropriate and cited with a study note. Cells were removed from the incubator and the growth media was removed by aspiration and 100 µL of warm sample dilution media was then transferred to the cells, in triplicate. Plates were incubated at 37°C ± 2°C, 5% ± 1% CO2, ≥70% relative humidity for 120 h. After 120 h plates were removed from the incubator and scored for the presence of cytopathic effects (CPE) using an inverted microscope. If CPE was present the wells were scored as “+” and if there was no presence of CPE the wells were scored as “-”.

RT-qPCR Viral Titer Analysis of Lung Tissue: One-step RT-qPCR was performed using isolated RNA from left lung tissue samples to estimate viral copy number. Briefly, RNA was extracted from the lung tissue samples that were stored in RNA/DNA Shield using the Quick-RNA Viral Kit (Zymo Research). Following the manufacturer’s protocol, RT-qPCR analysis was performed using the BlazeTaq Probe One-Step RT-qPCR Kit (GeneCopoeia; Rockville, MD) using isolated RNA as template. The following RTqPCR cycling conditions were used: 50°C for 15 min (RT), 95°C for 2 min (denature), then 40 cycles of 10 s at 95°C followed by 45 s at 62°C (extension). The following primers and probe were used for SARS-CoV-2 detection:

Primers:

2019-nCoV_N1-F:

5′-GAC​CCC​AAA​ATC​AGC​GAA​AT-3′

2019-nCoV_N1-R:

5′-TCT​GGT​TAC​TGC​CAG​TTG​AAT​CTG -3′

Probe:

2019-nCoV_N1-P:

5′-FAM-ACCCCGCATTACGTTTGGTGGACC-BHQ1-3′

For estimating viral copy number, samples were compared against a standard curve with synthetic RNA, this particular reagent (BlazeTaq) produces a copy number calculation ∼1 log higher in comparison to copy number calculation with other suppliers (i.e. Bio-Rad reagent, iTaq) however, for samples, the copy number is comparable.

Data Analysis: Descriptive statistics (mean and standard deviation) were calculated for clinical chemistry data using the ToxData^®^ system. Descriptive statistics for body weight, body weight change (from baseline), and viral titer data were calculated using Microsoft Excel.

### 4.2 Results

Mebendazole alone resulted in a 44.2% reduction respectively in lung viral load compared to non-treated placebo control (Table 7). There were no significant differences in body weight (data not shown).

**TABLE 7:** Lung RT-qPCR data for SARS-CoV-2 virus following 6 days of treatment in K18-hACE2 mice orally administered with Mebendazole) (50 mg/kg) once daily for 6 consecutive days.

Treatment group	N	Lung RT-qPCR mean ± SD(×10^7^)	% Reduction compared to control
Untreated control	7	7.81 ± 6.0	—
Mebendazole	7	4.36 ± 2.2	44.2%

On Day 6, serum biochemistry markers were assessed for liver or renal injury and no significant changes in ALT, AST, or BUN levels were observed for mebendazole-treated mice, suggesting no significant toxicities following drug treatments. Furthermore, no abnormal histopathological findings in tissues and samples collected (nasal turbinates, stomach, jejunum, ileum, colon, femur, bronchial lymph node, and right lung) at necropsy were observed, supporting a favorable safety profile of mebendazole (data not shown).

ALT, AST, and BUN, as a measure of drug hepatic and renal toxicity were measured on Study Day 6. ALT (normal range 17–77 U/L). However, all other levels were within acceptable reference values for mouse serum ALT, AST (54–298 U/L), and BUN (8–33 mg/dL) values. As treatment was up to Day 6, values suggest that the acute treatment of mebendazole did not result in liver or renal injury.

## 5 Discussion

The identification and deployment of cost-effective, safe, and accessible therapeutics to treat COVID-19 within LMIC (Low- and Middle-Income Countries) communities remains a challenge (Hancock et al., 2016; Maxwell et al., 2021). Using our AI platform in a short time frame we have identified potential candidates that could be potentially used for the prevention and treatment of COVID-19 particularly in patients with mild to moderate symptoms of the disease (Naderi et al., 2016; Liu et al., 2017; Pu et al., 2019; Bess et al., 2022).

In our AI analysis, several compounds were identified to potentially inhibit the entry, fusion, and replication of SARS-CoV-2. Based on this analysis several compounds were tested in lung cancer cells infected with the virus to determine potential antiviral activity and non-toxic doses. One such drug widely used in the developing world to treat pinworm that is cost-effective, safe, and accessible is Mebendazole. Our cell results and subsequent animal studies suggested that Mebendazole could be an effective oral therapeutic for COVID-19.

Mebendazole is approved in the United States, Canada and Europe for treatment of gastrointestinal infections caused by helminths (parasitic worms) (Janssen, 2019b, 2021). It inhibits microtubules in the parasites, and this mechanism of action may afford some antiviral effects against SARS-CoV-2 by obstructing the trafficking of SARS-CoV-2 and viral structural proteins within the cell, resulting in decreased viral entry, viral replication, and assembly and egress (exocytosis) of newly made SARS-CoV-2 virions from the cell (Figure 1).

Importantly, mebendazole is a potent inhibitor of several tyrosine protein kinases, including mitogen-activated-protein-kinase 14 (MAPK14) and ABL1 (Abelson tyrosine protein kinase 1) (Ariey-Bonnet et al., 2020). Based on strong evidence from the literature, MAPK14 inhibition is thought to decrease SARS-CoV-2 replication and significantly reduce the hyper-inflammatory response associated with SARS-CoV-2 infection (Bouhaddou et al., 2020; Klann et al., 2020). Based on this dual mechanism of action (microtubule and MAPK14 inhibition), mebendazole may exhibit both antiviral and anti-inflammatory effects.

However, based on the low oral bioavailability of mebendazole, it was likely not absorbed in sufficient quantities to affect concentrations of virus in the lungs (Kuhlmann and Fleckenstein, 2017). Indeed, its low systemic bioavailability accounts for its relative lack of activity in extraintestinal infections. However, mebendazole has been used to treat human alveolar echinococcosis, a lethal pulmonary helminthic infection (Sasaki et al., 2002). Although echinococcosis requires long-term treatment with mebendazole, the incidence of severe side effects is low. Daily doses of 50 mg/kg (for up to 30 days) together with other drugs to improve its absorption was observed to increase the efficacy of mebendazole in the context of pulmonary infections (Shcherbakov et al., 1993; Simbulan-Rosenthal et al., 2017; Ariey-Bonnet et al., 2020). Because its delivery to human peripheral lung tissue has few or no side effects, mebendazole is a promising antiviral therapeutic against SARS-CoV-2 at the planned clinical dose based on the AI analysis and preliminary *in vivo* data. While our findings appear promising, we acknowledge a limitation in our pilot animal study: it was conducted using only a single dose within an acute model of COVID-19. Further in-depth dose response studies in milder COVID-19 models are necessary to more comprehensively assess the therapeutic potential of mebendazole.

Another drug that showed promising antiviral activity in our preliminary cell studies was Imatinib (data not shown). Imatinib is approved in the United States, Canada and Europe for treatment of adult and pediatric patients with Philadelphia chromosome positive chronic myeloid leukemia (Ph+ CML) and acute lymphoblastic leukemia (Ph+ ALL) (Dalziel et al., 2005; Peng et al., 2005; Talarico et al., 2005; Kantarjian et al., 2009; Abou Dalle et al., 2019). Imatinib is a strong anti-inflammatory agent that, like mebendazole, may decrease the hyper-inflammatory response associated with SARS-CoV-2 infection and prevent severe tissue damage. Through inhibition of ABL1, which has been shown to be involved in actin polymerization/formation of actin tails and cell-to-cell transmission of new virions, imatinib may also demonstrate direct antiviral activity (Reeves et al., 2011; García et al., 2012; Gancheva et al., 2013).

However, several studies from the literature were identified in which imatinib inhibited SARS-CoV-2, SARS-CoV-1 and MERS-CoV replication at concentrations of ∼10 µM (ASSAAD and ASSAAD-KHALIL, 2020; ClinicalTrials, 2020; Zhao et al., 2020) In parallel experiments in one of the studies, imatinib did not show an inhibitory effect on SARS-CoV-2 entry/infection at concentrations up to 10 μM (Zhao et al., 2020). The conclusion from these reports is that imatinib is not a potent inhibitor of viral entry but at clinically achievable dose ranges (400–800 mg/day), imatinib may have some effect on SARS-CoV-2 replication. Though imatinib is not a potent antiviral agent, it still holds promise for the treatment of COVID-19 through its anti-inflammatory effects (i.e., reduced cytokine-induced inflammatory response and tissue injury).

In an era where novel viruses can emerge rapidly and spread globally within months, a tool like eVir becomes indispensable. Such a framework provides a proactive approach to viral threats, rather than a reactionary one. By being virus agnostic, eVir offers a foundational platform upon which specific viral data can be integrated and analyzed quickly. This speed and adaptability are crucial for early intervention and the development of effective treatments. Furthermore, as research advances, tools that can swiftly assimilate and make sense of vast datasets become paramount. eVir not only helps in identifying potential therapeutic interventions for current viral outbreaks but also serves as a research scaffold for the scientific community. Researchers can build upon this framework, refining algorithms, adding newer datasets, and improving prediction accuracy. This continuous evolution ensures that we remain one step ahead, better prepared and more informed, for any future viral challenges.

Our future direction will pivot toward testing other predicted compounds that eVir flagged with high confidence. While AI has been used in diverse domains, its widespread use in drug discovery has been hindered by the uncertainty in the predictions. In future, we plan to quantify the uncertainty associated with predictions from engines like eVir in a formal reasoning framework. While AI drug discovery platforms have begun to proliferate, their efficacy varies, and some holistic systemic details of the treatments are occasionally overlooked. One fascinating direction is the results of the MLP/SNet models and its possible correlation with post-acute sequelae of SARS-CoV-2 infection (often termed ‘Long Covid’) remain critical areas of exploration (Lazebnik, 2021). As the fight against COVID-19 progresses, AI-integrated drug discovery promises to be a beacon in uncovering new therapeutic vistas, especially within LMIC communities.

## Data Availability

The raw data supporting the conclusion of this article will be made available by the authors, without undue reservation.
